# Antibody‐based methods for the measurement of α‐synuclein concentration in human cerebrospinal fluid – method comparison and round robin study

**DOI:** 10.1111/jnc.14569

**Published:** 2018-11-13

**Authors:** Brit Mollenhauer, Frederick DuBois Bowman, Daniel Drake, Jimmy Duong, Kaj Blennow, Omar El‐Agnaf, Leslie M. Shaw, Jennifer Masucci, Peggy Taylor, Robert M. Umek, Jill M. Dunty, Chris L. Smith, Erik Stoops, Hugo Vanderstichele, Adrian W. Schmid, Marc Moniatte, Jing Zhang, Niels Kruse, Hilal A. Lashuel, Charlotte Teunissen, Tanja Schubert, Kuldip D. Dave, Samantha J. Hutten, Henrik Zetterberg

**Affiliations:** ^1^ Paracelsus‐Elena‐Klinik Kassel Germany; ^2^ Department of Neurology University Medical Center Goettingen Germany; ^3^ Department of Biostatistics Columbia University Mailman School of Public Health New York City New York USA; ^4^ Department of Psychiatry and Neurochemistry the Sahlgrenska Academy at the University of Gothenburg Mölndal Sweden; ^5^ Clinical Neurochemistry Laboratory Sahlgrenska University Hospital Mölndal Sweden; ^6^ Neurological Disorders Research Center Qatar Biomedical Research Institute (QBRI), and College of Science and Engineering HBKU, Education City Qatar Foundation Doha Qatar; ^7^ Department of Pathology & Laboratory Medicine and Center for Neurodegenerative Disease Research Institute on Aging University of Pennsylvania Philadelphia PA USA; ^8^ BioLegend Dedham MA USA; ^9^ Meso Scale Discovery Gaithersburg MD USA; ^10^ ADx NeuroSciences Gent Belgium; ^11^ Proteomics Core Facility Ecole Polytechnique Fédérale de Lausanne (EPFL) Lausanne Switzerland; ^12^ University of Washington Seattle WA USA; ^13^ Institute of Neuropathology University Medical Center Goettingen Germany; ^14^ Laboratory of Molecular and Chemical Biology of Neurodegeneration Brain Mind Institute Institute of Physics of Biological Systems Ecole Polytechnique Fédérale de Lausanne (EPFL) CH‐1015 Lausanne Switzerland; ^15^ VU University Medical Center Amsterdam the Netherlands; ^16^ Bioclinica Lyon France; ^17^ Michael J. Fox Foundation for Parkinson's Research New York City New York USA; ^18^ Department of Molecular Neuroscience UCL Institute of Neurology Queens Square London UK; ^19^ UK Dementia Research Institute at UCL London UK

**Keywords:** biomarker, cerebrospinal fluid, enzyme‐linked immunoabsorbent assay, mass spectrometry, round robin, α‐synuclein

## Abstract

α‐Synuclein is the major component of Lewy bodies and a candidate biomarker for neurodegenerative diseases in which Lewy bodies are common, including Parkinson's disease and dementia with Lewy bodies. A large body of literature suggests that these disorders are characterized by reduced concentrations of α‐synuclein in cerebrospinal fluid (CSF), with overlapping concentrations compared to healthy controls and variability across studies. Several reasons can account for this variability, including technical ones, such as inter‐assay and inter‐laboratory variation (reproducibility). We compared four immunochemical methods for the quantification of α‐synuclein concentration in 50 unique CSF samples. All methods were designed to capture most of the existing α‐synuclein forms in CSF (‘total’ α‐synuclein). Each of the four methods showed high analytical precision, excellent correlation between laboratories (*R* ^2^ 0.83–0.99), and good correlation with each other (*R* ^2^ 0.64–0.93), although the slopes of the regression lines were different between the four immunoassays. The use of common reference CSF samples decreased the differences in α‐synuclein concentration between detection methods and technologies. Pilot data on an immunoprecipitation mass spectrometry (IP‐MS) method is also presented. Our results suggest that the four immunochemical methods and the IP‐MS method measure similar forms of α‐synuclein and that a common reference material would allow harmonization of results between immunoassays.

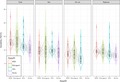

**Abbreviations used**
AD: Alzheimer's disease
CJD: Creutzfeldt–Jakob disease
CSF: cerebrospinal fluid
DLB: dementia with Lewy bodies
HGB: hemoglobin
MSA: multiple system atrophy
OLS: ordinary least squares
PD: Parkinson's disease
PPMI: Parkinson's Progression Marker Initiative
TSQ: triple quadrupole mass spectrometer
VCA: variance component analysis

α‐Synuclein is a mainly pre‐synaptic protein involved in synaptic vesicle release and the major component of aggregates that are characteristic inclusions of Parkinson's disease (PD), dementia with Lewy bodies, and multiple system atrophy (Mollenhauer *et al*. 2010). In PD and other α‐synuclein‐related disorders, cerebrospinal fluid (CSF) α‐synuclein concentrations are typically 10–15% lower than in healthy controls (Mollenhauer *et al*. 2011; Hall *et al*. 2012; Kapaki *et al*. 2013; Eusebi *et al*. 2017). However, CSF α‐synuclein concentrations are increased in Alzheimer's disease (AD), and even more pronounced in Creutzfeldt–Jakob disease, suggesting that there may be a competition between aggregation of α‐synuclein into Lewy bodies (LBs) and release of the protein from degenerating synapses (Ohrfelt *et al*. 2009; Mollenhauer *et al*. 2011; Tateno *et al*. 2012; Wennstrom *et al*. 2013; Slaets *et al*. 2014; Llorens *et al*. 2016), making the data complex to interpret.

Most currently available assays for α‐synuclein have been designed to quantify total amounts of the protein and not disease‐specific isoforms or the post‐translationally modified and aggregated forms of the protein. There are some preliminary reports on increased CSF concentrations of α‐synuclein oligomers in CSF from PD patients (Tokuda *et al*. 2010; Hansson *et al*. 2014). Recently, sensitive assays that detect and amplify the biochemical signal of putative seeds of α‐synuclein oligomers in CSF were published (Fairfoul *et al*. 2016; Shahnawaz *et al*. 2017; Groveman *et al*. 2018).

In spite of the uncertainty regarding the diagnostic performance of CSF α‐synuclein, because of an overlap in concentrations and discrepancies between study populations, there are several clinical trials in which α‐synuclein expression, release, and/or aggregation are targeted (Valera and Masliah 2013). In these trials, reliable methods to quantify α‐synuclein are desired, not only for the purposes of assessing target engagement and pharmacodynamic responses, but also potentially for patient stratification (Toledo *et al*. 2013). Such assays could also be used to standardize concentrations of pathology‐enriched forms of α‐synuclein (e.g., phosphorylated forms, oligomers).

Here, we assessed the analytical characteristics of four different immunoassays (with different antibody pairs targeting different regions) measuring CSF α‐synuclein, and determined how the assays correlated with each other using multiple aliquots of 50 CSF samples collected according to standard operating procedures (Kang *et al*. 2013). Pilot data using an immunoprecipitation mass spectrometry (IP‐MS) method are also presented. We demonstrate good correlation between the different immunoassays.

## Methods

Multiple aliquots of 50 unique CSF samples representing α‐synuclein concentrations spanning the clinically relevant range of α‐synuclein were collected at the Paracelsus‐Elena‐Klinik, Kassel, Germany (IRB number: UMG 9/7/04) and the University of Washington, Seattle, USA (IRB number: 44084) in accordance with the Michael J. Fox Foundation's Parkinson's Progression Markers Initiative protocols (http://ppmi-info.org/study-design) (Kang *et al*. 2013), frozen at −80°C and distributed to the participating laboratories on dry ice.

A power calculation by general linear mixed models accounting for correlated samples across different laboratories (type‐I error level set to α = 0.05) was performed. A sample size of 50 would yield a power exceeding 0.90 to detect a difference in α‐synuclein concentration of 10 pg/mL. Accounting for sample loss because of quality control assessments, a sample size of 40 would yield power of 0.85 to detect difference of 10 pg/mL.

Donors of samples had a mean age of 69.8 years ±6.6 (standard deviation) (27 men, 23 women) and had a clinical diagnosis of PD (*n* = 22) according to UK Brain Bank Criteria, other neurological movement disorders (such as normal pressure hydrocephalus, multiple system atrophy or progressive supranuclear palsy; *n* = 13) and healthy controls (*n* = 15). The study was not pre‐registered. No randomization was performed. All people involved in this study besides SJH and the statistical team were blinded as to the diagnoses. The samples size was determined by feasibility. See above for power calculations. CSF hemoglobin (HGB) was measured centrally using an enzyme‐linked immunosorbent assay from Bethyl Laboratories (Cat. No. E88‐134) according to the manufacturer's instructions. The vials used for the ADx NeuroSciences ELISA were generated in a second round of fractionating using the same protocol as for the first round. Tubes from the first and second round were compared for reactivity (*n* = 50) and homogeneity (limited number; *n* = 5) and found equivalent in concentration and homogeneity.

Four different immunoassays were compared: the Elecsys^®^ Total α‐Synuclein Prototype Assay (Roche Diagnostics, Penzberg, Germany; not commercially available), the MSD U‐PLEX^®^ Human α‐Synuclein Kit (Meso Scale Discovery, Rockville, MD, USA; Cat. No. K151WKK‐1), the BioLegend α‐Synuclein Immunoassay (BioLegend, San Diego, CA, USA; Cat. No. 844101), and the α‐Synuclein Prototype Immunoassay from ADx (ADx NeuroSciences, Gent, Belgium, available through Euroimmun, Lübeck, Germany; Cat. No. EQ 6545‐9601‐L). All assays were designed with a combination of two monoclonal antibodies recognizing different α‐synuclein epitopes and used different detection technologies (summarized in Table 1).

**Table 1:** Technology characteristics of the four immunochemical methods that were used to quantify CSF α‐synuclein

	Roche	MSD	BioLegend	ADx
Technology				
Sandwich immunoassay	+	+	+	+
Detection	ECL	ECL	Luminescence	Absorbance‐based
Automation	+	−	−	−
Antibodies				
Capture antibody (epitope)	No information	No information	aSyn (118–122)	ADx301 (no information)
Detector mAb (epitope)	No information	No information	aSyn (103–108)	ADx302 (no information)
Calibrator				
Type	Recombinant	Recombinant	Recombinant	Recombinant

ECL = electrochemiluminescence; rec. = recombinant.

The Elecsys^®^ prototype assay is based on two anti‐α‐synuclein antibodies and utilizes the electrochemiluminiscence detection technology. The assay has been developed for CSF analysis on the Roche Elecsys^®^ cobas e411 and e601 platforms, which are fully automated instruments (Bittner *et al*. 2016). The consistency of automated versus manual measurement on β‐amyloid 1‐42 was published and not subject of this current study (Bittner *et al*. 2016). Only one kit lot was produced by Roche for this study. The MSD kit is a sandwich immunoassay with a biotinylated capture antibody and a SULFO‐TAG™‐labeled detection antibody specific for α‐synuclein, which is read by MSD instruments with electrochemiluminiscence detection technology (for more details, see: https://www.mesoscale.com/products/human-alpha-synuclein-kit-k151tgd). The BioLegend assay is a sandwich type immunoassay with luminescence readout developed using the same anti‐α‐synuclein antibodies as those described by Mollenhauer *et al*. (2011) (capture and detection epitopes in the C‐terminal domain of α‐synuclein). The kit can be read by any luminescence reader and does not rely on a particular instrument but variation because of different luminescence readers has been described (Kruse *et al*. 2015). The following luminometers were used for the BioLegend assay for this study: Glomax Multi detection system (Promega; University of Washington), Victor ×4 (PerkinElmer, Gothenburg), BioTek Synergy 2 (BioTek; BioLegend), and Glomax 96 Microplate Luminometer (Promega; Goettingen) (for more details on the assay, see: https://www.biolegend.com/en-us/products/legend-max-human-alpha-synuclein-elisa-kit-with-pre-coated-plate-11210). The ADx α‐synuclein kit is an absorbance‐based sandwich ELISA with a readout at OD_450–600 nm_. α‐Synuclein is captured by a C‐terminal specific monoclonal antibody (ADx301) and incubated for 3 h at room temperature with a biotinylated C‐terminal‐specific antibody (ADx302). Twenty‐five μL of undiluted CSF is used. Subsequently, peroxidase‐labeled streptavidin is added. 3,3′,5,5′‐Tetramethylbenzidine substrate is used to generate a colored product that can be read in a conventional microplate reader. The assay can be run manually or on an open automated system.

All assays used recombinant α‐synuclein as the calibrator, but the production and value assignments of the calibrators were performed by each vendor individually. Assay characteristics are specified in Table 1. In this study, different satellite laboratories were selected according to the equipment and available expertise.

The classical immunoassays were also compared to results obtained in one laboratory using immunoprecipitation combined to mass spectrometry (IP‐MS). The method is described elsewhere (Schmid *et al*. 2018 in preparation). In brief, human CSF samples (1 mL) were immunoprecipitated overnight using a rabbit polyclonal anti‐α‐synuclein antibody (FL‐140; Santa Cruz, USA; Cat. No. sc‐10717). The following day, beads and antibody‐bound α‐synuclein complexes were eluted and spiked with an accurately quantified (by amino acid analysis) heavy labeled (^15^N), recombinant human α‐synuclein reference protein standard (Fauvet *et al*. 2012). The immunoprecipitated samples were then subjected to trypsin (Trypsin Mass Gold Spectrometry Grade, Cat. No. V5280, Promega, Switzerland) and Glu‐C proteolysis (Glu‐C Sequencing Grade, Cat. No. V1651, Promega, Switzerland) as follows. Maximal α‐synuclein sequence coverage was achieved through a combined proteolysis protocol where the immunoprecipitated CSF samples were digested overnight with trypsin protease for mainly N‐terminal sequence coverage, followed by an 8 hr sequential digestion with Glu‐C protease for full C‐terminal sequence coverage. This dual proteolytic approach proved to be highly reproducible, resulting in an α‐synuclein protein sequence coverage of ≥ 90%. The proteolytic efficiency (estimated at ≥ 95%) was routinely monitored by measuring the completion of the dual trypsin and Glu‐C cleavage product of the N‐terminal peptide of residues G84‐K96 as compared to the tryptic peptide of residues T81‐K96/K97. The processed CSF samples were analyzed by nano‐liquid chromatography (nano‐Acquity, Waters, Milford, MA, USA) coupled with multiple reaction monitoring (MRM) with optimized collision energies, using a triple quadrupole mass spectrometer (TSQ Vantage extended mass range; EMR, Cat. No. TSQ4800, Thermo Fisher Scientific, Waltham, MA, USA). The lower limit of quantification (LLOQ) (using IP&MS) was defined as the lowest α‐synuclein protein concentration with a coefficient of variation (CV) of ≤ 20% and was estimated at ≤ 20 pg/mL (full‐length protein) using the N‐terminal fragment of residues G84‐K96 and, ≤ 50 pg/mL (full‐length protein) using the C‐terminal fragment of residues G106‐E114. The limit of detection was defined as a signal‐to‐noise ratio of 3 and was estimated at ≤ 10 pg/mL and ≤ 25 pg/mL (full‐length protein) using the N‐ and C‐terminal peptides, respectively. The analytical (LC‐MRM) intra‐ and inter‐assay CVs were determined using heavy labeled (^15^N) α‐synuclein digests and ranged between ≤ 10% and ≤ 15%, respectively. The acquired MRM raw files were analyzed using Skyline software v.3.6.0 (MacCoss Laboratory, University of Washington, USA) and peak identification was verified manually to rule out any possible noise discrepancies.

Fifty CSF samples were analyzed in duplicates on each assay platform by the ‘originating laboratory’ (the lab where the assay was developed) and 3–4 ‘satellite laboratories’ that were equipped with the necessary technology platforms and technical expertise to run the assay. Correlations of each laboratory's kit‐lot‐averaged α‐synuclein concentrations with the intra‐assay average were analyzed. In addition, the correlations between CSF α‐synuclein concentrations for each method measured at the originating laboratory were compared. For these comparisons, concentrations were averaged across replicates and kit lots.

Finally, a pilot commutability study was performed: two distinct references were considered: the immunoassay concentrations themselves, averaged across the assays to avoid any bias associated with favoring one assay over the others; and the mass spectrometer concentrations measured by the IP‐MS procedure. Various regression techniques were used to derive assay‐specific affine transformations relating immunoassay concentration values to a given reference.

## Statistical methods

First, a basic quality control screening at the replicate level was performed: if any replicate concentration fell outside of the assay's specified quantitative range, the measurement for that sample was excluded (which was the case once). If no replicate‐level concentrations were available for a sample on a particular run, the concentration for that sample was deemed missing. Otherwise, the sample concentration was computed as the mean of the non‐missing replicate‐level concentrations. If the run of a sample resulted in more than one valid replicate‐level measurement, then %CV for that sample was computed as well. No sample measurements were excluded because of excessive variability. Also, sample AL19 was determined to have excessive HGB concentration of 1971 ng/mL and was therefore excluded in the analysis. Experimental errors (e.g., single well testing) are reported.

Figures S1–4 plot show variability (%CV, along y) against sample ID (along x) for the 49 valid samples. The values are segregated by site and kit lot combination. Note that some samples have only one valid replicate and therefore do not have an associated variability measure.

Following quality control assessments, the replicates of the accepted samples were averaged, leaving one sample α‐synuclein value per assay, site, and kit lot. The variability in the resulting concentrations was examined from two aspects: kit lot variability per site and assay; and site variability per kit lot and assay. Subsequently, the kit lot values were averaged for each assay and site. The resulting averages allowed the comparison of inter‐site variability within each assay (see ranges specified in the next section), as well as visualization of differences in correlations between the sites. For each assay, a sample's average concentration was compared across sites and this value was used as a reference against which to examine each site's concentration individually.

In addition to the stepwise examination of the variability outlined above, variance component analysis (VCA) was performed on each assay's sample concentrations. VCA partitions the total variance associated with a particular sample's measured concentrations into components attributed to distinct sources of variability. VCA was performed at the assay level to assess the relative degree of variability associated with replicate, kit lot, and site. In addition, VCA was used at the site level to facilitate the comparison of kit lot and replicate variance components within assays.

The remaining analyses focus on the kit lot‐averaged samples from each assay's originating laboratory. For all four originating sites, a Kolmogorov–Smirnov goodness of fit test failed to reject the null hypothesis that the data are normal. We note, however, that all reported p‐values and confidence intervals in subsequent analyses were determined via non‐parametric methods and do not rely on the data's distribution. A visualization of the kit lot‐averaged concentrations from each assay's originating site (Figure S5) identified one mild outlier above the threshold defined as 1.5 times interquartile ratio plus the third‐quartile. However, a Grubbs’ test for outliers failed to reject the null hypothesis that there are no outliers for all four assays.

To assess method equivalence, we considered two possible references: harmonization to average immunoassay concentrations; and harmonization to the independent source provided by the IP‐MS estimates. With both references, we explored harmonization in the context of affine transformations of the immunoassay concentrations to the reference. We bracketed harmonization performance by a) fitting the harmonization transforms to a single representative sample; and b) regressing all 49 samples against the reference, via ordinary least squares or Passing‐Bablok, to derive the transformation of each individual assay to the reference. In the first case, the transform amounted to a simple scale factor; the latter case allowed the estimation of not just a scale factor but an intercept adjustment as well. The pre‐ and post‐harmonization inter‐assay associations were evaluated both graphically, by examining the bias relative to the reference samples; and analytically, via Passing‐Bablok regression, in tandem with cumulative sum (CUSUM) linearity and Wald–Wolfowitz runs tests, and Kendall's tau to assess degree of association.

The statistical analyses were carried out with R: version 3.5.0, VCA package: version 1.3.3 and mcr package: version 1.2.1.

## Results

### Sample preparation

Homogeneity of aliquot preparation was verified in the facilities of ADx by testing four aliquots (concentration range: 1059–3049 pg/mL) of five CSF samples in duplicate. The inter‐vial variability ranged from 2.9 to 6.0%. Repeatability (replicate test, one sample, *n* = 16) was good with a median %CV (p25, p75) amounted to 7.8 (4.6–9.6). The correlation between concentrations in the two sample sets is excellent (*R* ^2^=0.9652; y = 1.078x – 68). The HGB levels ranged from 0.45 to 1971 ng/mL with a mean value and standard deviation of 50.76 ± 275.1 ng/mL. The one sample with 1971 ng/mL was considered significantly blood‐contaminated and excluded from the study.

### CSF α‐synuclein replicate correlations

Each site measured two replicates of each CSF sample's α‐synuclein concentration. For the Roche assay, there was one kit lot and four laboratories (including originating and satellite laboratories and two devices: e411 in Gothenburg and e601 in all other laboratories) (Figure S1a–d). For the MSD assay, there were three kit lots and four laboratories (Figure S2a–l). Also for the BioLegend assay, there were three kit lots and four laboratories (Figure S3a–l). For ADx, two kit lots were used to perform the analysis in five laboratories (Figure S4a–j). For most assays, repeatability results (variability in measurement replicates) were excellent, showing the maximum 95th percentile CV of 12.1% on the MSD platform, 13.1% for BioLegend, 17.1% for ADx, and 17.5% for Roche's system.

### CSF α‐synuclein site‐to‐site variability and correlations between sites for each assay

**Figure 1:**
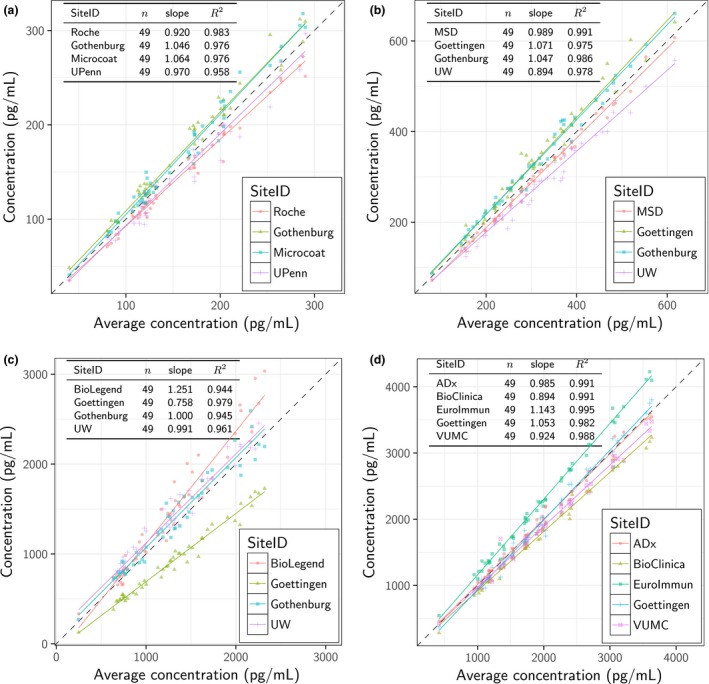
Scatter plots for MSD (a), BioLegend (b), ADx (c), and Roche (d) CSF α‐synuclein assays. On the *y*‐axis of each panel, each sample's concentration per site (i.e., averaged across replicates and kit lots) is shown. The corresponding average concentration across all sites is given on the x axis. The results from linear regression of each site's concentrations against the average are shown, with corresponding number of the 49 samples, slope, and coefficient of determination (*R* ^2^). The dotted line indicates x = y.

Inter‐laboratory comparisons for each CSF α‐synuclein assay are shown in Fig. 1. The Roche, MSD, and ADx assays showed excellent inter‐laboratory correlations. The mean inter‐laboratory coefficients of variation were 10.2%, whereas the BioLegend assay had slightly larger variation between laboratories (inter‐laboratory CV 15.8–34.9%). The higher site and total variability with the BioLegend assay is driven by measurements from one laboratory using a plate reader that was off in calibration (Kruse *et al*. 2015). If that laboratory is removed, BioLegend's variance components return to levels commensurate with the other assays (inter‐laboratory CV 1.3–20.5%).

**Figure 2:**
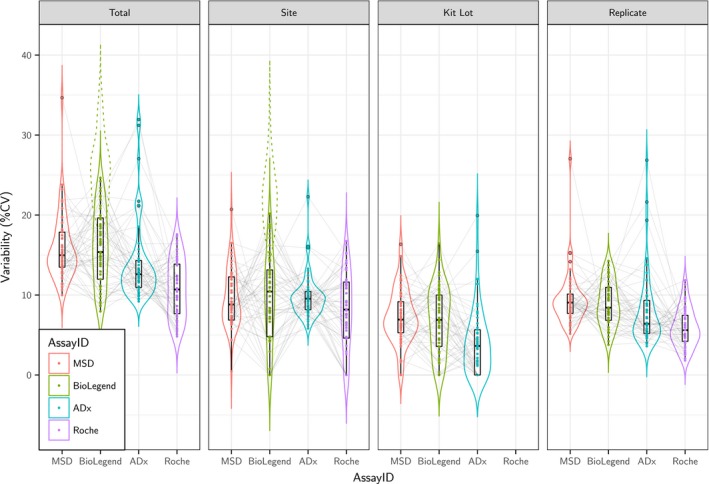
Variance components (expressed as % CV) per assay. Variance component analysis partitions the total variance of all assays associated with each sample's measurements (*n *=* *49) into site components, kit lot, and replicate. The distribution of the components by assay are highlighted by the box and violin plots. Individual samples are joined by the light gray lines; no one sample appears to have elevated variance components across assays. The impact of Goettingen's unusual results in the BioLegend assay has been removed from the analysis, leaving the BioLegend assay with variance components comparable to the other assays. (The total and site variance components with Goettingen included are shown by the dotted lines.) Note that Roche measured their samples with the equivalent of just one kit lot; hence, that assay has no kit lot variance component.

Figure 2 illustrates the results of the VCA, indicating the variance components for each sample and assay along with the mean component level across the 49 samples for each assay. Table 2 quantifies these levels in terms of %CV and percent of total variability (% Total), both for each assay in full as well as for the individual sites associated with the assay. As above, the mean total variability in BioLegend was larger (%CV of 25.6%) than for Roche, MSD, and ADx (ranging from %CV of 10.8% to 15.9%). However, upon removal of Goettingen from the BioLegend data (plate reader off calibration), the mean total variability dropped to levels on par with the other assays (%CV of 15.9%). Considering relative proportions of variability (%Total), the site component took up the largest share of variability (approximately 40% of total for MSD and BioLegend with Goettingen excluded, 55% of total for ADx, and 60% of total for Roche). Then followed the replicate component (slightly more than 35% of total for MSD and BioLegend, slightly < 35% of total for ADx, and 40% of total for Roche), and finally the kit lot component (about 25% of total for both MSD and BioLegend, and < 15% of total for ADx). Given the lack of kit lot component for Roche, one should not compare its overall %CV with the other assays; similarly, Roche's lack of kit lot component resulted in inflated %Total values for the other two components.

**Table 2:** Mean variance components expressed as %CV and percent of total variability (%Total) for each assay in full (ALL), as well for each affiliated site. Note that Roche measured α‐synuclein concentrations with just one kit lot; hence, no kit lot component of variability is available for that assay. Note also that one of BioLegend's satellite laboratories had a miscalibrated plate, as indicated by the asterisk. An additional row for BioLegend (ALL but one) presents the mean variance components with that laboratory removed

AssayID	SiteID	Total		Replicate		Kit Lot		Site	
		%CV	%Total	%CV	%Total	%CV	%Total	%CV	%Total
MSD	ALL	15.93	100	9.47	37.16	7.27	24.59	9.61	38.25
	Satellite lab 1	15.66	100	4.76	22.66	13.83	77.34		
	Satellite lab 2	11.72	100	2.64	10.77	11.27	89.23		
	Satellite lab 3	8.01	100	2.62	20.39	7.37	79.61		
	Satellite lab 4	10.13	100	2.99	19.33	9.40	80.67		
BioLegend	ALL	25.56	100	9.91	16.10	5.70	6.98	22.37	76.92
BioLegend	ALL but one	15.93	100	8.93	36.67	6.89	23.75	9.64	39.58
	Satellite lab 1	12.88	100	4.67	30.65	10.98	69.35		
	Satellite lab 2*	12.72	100	4.30	15.94	11.63	84.06		
	Satellite lab 3	10.75	100	4.75	32.03	8.62	67.97		
	Satellite lab 4	11.78	100	3.20	13.46	11.09	86.54		
ADx	ALL	14.08	100	8.07	32.73	4.08	12.60	9.81	54.67
	Satellite lab 1	8.71	100	5.48	58.49	5.24	41.51		
	Satellite lab 2	9.87	100	5.94	45.97	6.95	54.03		
	Satellite lab 3	6.28	100	4.22	57.78	3.34	42.22		
	Satellite lab 4	10.65	100	6.30	56.23	6.89	43.77		
	Satellite lab 5	7.96	100	3.81	40.73	6.09	59.27		
Roche	ALL	10.80	100	5.91	40.07	NA	NA	8.12	59.93
	Satellite lab 1	3.52	100	3.52	100	NA	NA		
	Satellite lab 2	4.61	100	4.61	100	NA	NA		
	Satellite lab 3	4.83	100	4.83	100	NA	NA		
	Satellite lab 4	6.28	100	6.28	100	NA	NA		

### Inter‐assay comparisons

**Figure 3:**
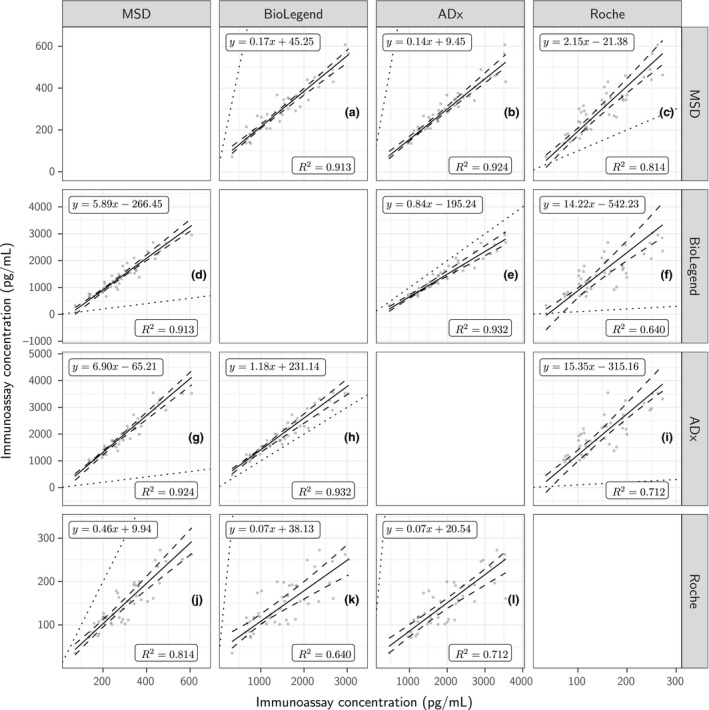
Relationship between the CSF α‐synuclein concentrations for MSD in y against BioLegend (a), ADx (b), and Roche (c) in x; for BioLegend in y against MSD (d), ADx (e), and Roche (f) in x; for ADx in y against MSD (g), BioLegend (h), and Roche (I) in x; and Roche in y against MSD (j), BioLegend (k), and ADx (l) in x. All concentrations of 49 samples measured at the originating laboratories (the laboratories that developed the respective assay) and have been averaged across replicates and kit lots.

The relationships between the CSF α‐synuclein concentrations derived using the Roche, MSD BioLegend, and ADx CSF α‐synuclein assays were examined using data from the originating laboratories (since these laboratories were where the assays had been developed, these were considered the best case scenario expert laboratories) (Fig. 3a–l). The results showed excellent correlations, but the slopes of the different linear regression lines confirmed that the assays were not standardized using the same reference material. We note that the coefficients of determination associated with comparisons to Roche were uniformly less than any of the other comparisons (largest Roche *R* ^2^=0.814 vs. smallest in the complement: *R* ^2^=0.913). The ADx and BioLegend methods gave higher concentrations than the MSD and Roche assays (334–3547 pg/mL, compared to 35.1–607 pg/mL).

### Quantification of CSF α‐synuclein by IP‐MS

**Figure 4:**
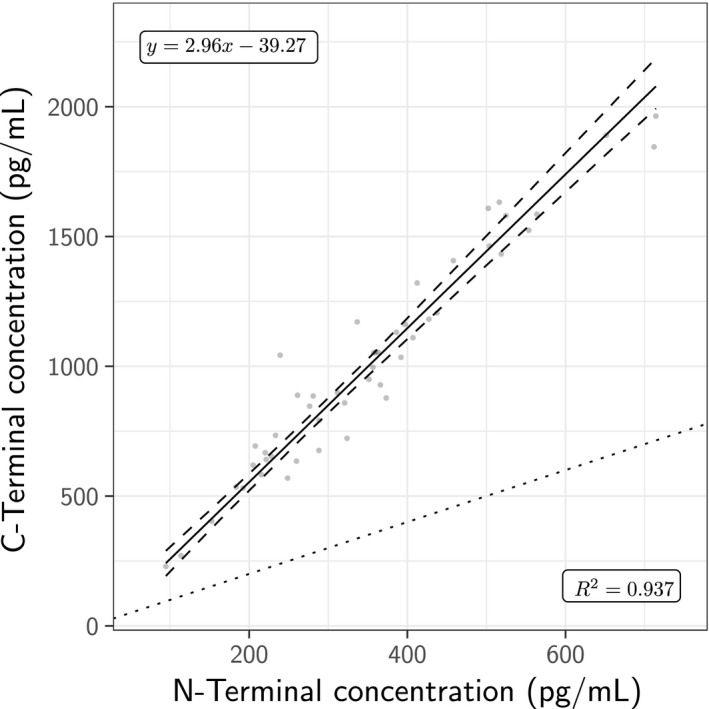
Association between average α‐synuclein concentrations determined by five N‐Terminal fragments and three C‐Terminal fragments from the IP‐MS mass spectrometry procedure. The dotted line indicates the identity y=x. Passing‐Bablok regression results are shown, with the estimate (solid line) and corresponding 95% confidence intervals (curved dashes).

We also used a newly developed IP‐MS method to quantify CSF α‐synuclein in all 50 samples in one laboratory. Concentrations ranged from 94.7 to 714 pg/mL for N‐terminal fragments and 229–1964 pg/mL for C‐terminal fragments. N‐ and C‐terminal fragments correlated strongly (Fig. 4).

### Pilot commutability study

The inter‐assay bias seen in Fig. 3a–l may be attributed to the fact that each kit used different calibrant preparations (=calibrator added to buffered solutions), the assay design, or the antibody‐pair selection. A common reference may be a critical tool to control for inter‐assay variability. Since at this time no reference material for CSF α‐synuclein exists, we considered two possible references: harmonization to average immunoassay concentrations; and harmonization to the independent source provided by the IP‐MS estimates.

### Harmonization to immunoassay reference

The reference in this section was computed by averaging α‐synuclein concentrations for each sample across the originating sites of the assays. Once averaged, we either focused on one particular sample (AL41or AL48, two samples with relatively high mean concentrations and low overall variability), or the whole set of samples, depending on the harmonization approach.

We present the harmonization procedure in three steps, illustrated in Figure S6:

- Compute the harmonization transform: Figure S6a plots the values for each assay (y) against the common reference (x). The color‐coded regression lines indicate the association between the samples based on the harmonization procedure used. (In the cases that rely on a single sample, the corresponding regression line passes through the origin and the reference sample.)
- Apply the harmonization transform: Figure S6b illustrates the result of the harmonization procedure. Note that since the harmonization transform differs per approach, the transformed points are not coincident. After harmonization, we performed Passing‐Bablok regression to assess the quality of the harmonization. In this figure, the regression lines indicate the result of the Passing‐Bablok regression on the harmonized points, *not* the transformed regression lines from neighboring Figure 9A (by construction, upon transformation those regression lines align with the identity x = y).
- Assess the harmonization transform: Figure S6C indicates the percent difference of each method's associated Passing‐Bablok regression line from the common reference.

Figure S6 assesses the harmonization procedure relative to the reference. Analytic characterization of the four transforms found improved harmonization using the Passing‐Bablok‐derived transformation (i.e., the Passing‐Bablok regression of the harmonized assays against the reference gave confidence intervals straddling zero and the slope confidence intervals straddling one).

In no case did the CUSUM linearity test nor the Wald‐Wolfowitz reject the null, indicating a degree of linearity between the reference and individual assays and only random fluctuations around the regression line. The smallest Kendall tau among the four assays was tau=0.631 for Roche.

Figures S7 and 8 illustrate the pairwise, post‐harmonization, comparison between assays. Figure S7 evaluates the pairwise association via Passing‐Bablok regression between the assays after harmonization, while Figure S8 presents the smoothed percent difference of one assay relative to another for the different harmonization approaches. Of the four transformations, Passing‐Bablok performed the best, with all but one assay pair deemed not harmonized; Roche vs. BioLegend failed both the CUSUM linearity test (*p* = 0.034) and the Wald‐Wolfowitz runs test (*p* = 0.027), and had the smallest tau (Table S1).

A mixed model analysis on the post‐harmonized concentrations was unable to detect a significant difference between any assay pairs.

### Harmonization to IP‐MS (C‐terminal) reference

A similar approach was taken using the average of three C‐terminal fragments of the IP‐MS concentrations, with the analogous Figures S9, 10, and 11. However, when using the IP‐MS values as the reference, the least squares‐based approach results in transformations that leave several samples of the various immunoassays with negative concentrations. Removal of the negative samples from consideration led to additional samples with negative concentrations in the subsequent re‐fit using ordinary least squares (OLS). As a consequence, no OLS‐based results are presented for the mass spectrometer reference case. The Passing‐Bablok‐based transformations resulted in one sample with negative concentration (AL25), and upon removal of this sample from the harmonization procedure, the results were stable: no further harmonized samples were negative.

Harmonization to the reference, again, was best using the Passing‐Bablok‐derived transformation, with only the Roche assay's intercept and slope 95% confidence intervals not straddling 0 and 1, respectively. In terms of pairwise comparison after harmonization based on the IP‐MS reference, however, the transform based on sample AL41 had the most pairwise assay combinations that were commutable: MSD vs. ADx and MSD vs. Roche.

## Discussion

α‐Synuclein is a key protein in the pathogenesis of PD and other diseases characterized by Lewy body pathology (Galasko 2017). The quantification of the protein in CSF has been proposed as a diagnostic biomarker for PD and other α‐synuclein‐related diseases, such as multiple system atrophy and dementia with Lewy bodies. Most studies show decreased levels of total α‐synuclein in CSF samples from PD patients compared to control samples, but discrepant findings and overlapping values have been a major limitation for the use of CSF α‐synuclein as a biomarker (Mollenhauer 2014). The protein as such is released from neurons into the extracellular space and its CSF concentration is increased in diseases such as AD and Creutzfeldt–Jakob disease with pronounced neurodegeneration (Ohrfelt *et al*. 2009; Mollenhauer *et al*. 2011; Tateno *et al*. 2012; Wennstrom *et al*. 2013; Slaets *et al*. 2014; Oeckl *et al*. 2016). Its CSF concentration may thus be influenced by competing processes. Sequestration into Lewy bodies or decreased intracellular release could lead to lower α‐synuclein in the CSF, whereas release from degenerating synapses/neurons could increase its CSF levels. α‐Synuclein is also highly abundant in peripheral blood, making artificial blood contamination during the lumbar puncture a potential confounder (Hong *et al*. 2010).

In spite of these problems, reliable and reproducible methods for the quantification of CSF total α‐synuclein are absolutely critical as target engagement markers in synuclein clinical trials, as well as for use in patient stratification/selection. It is also possible that pathology‐enriched or modified forms of α‐synuclein need to be related to a measure of ‘total’ α‐synuclein to increase their diagnostic performance, much like AD‐associated Aβ42 becomes a better senile plaque marker if related to Aβ40 (Janelidze *et al*. 2016; Pannee *et al*. 2016; Lewczuk *et al*. 2017).

In this study, we compared four ‘total’ α‐synuclein assays (meaning that the assays were not developed for any specific pathology‐related isoform of α‐synuclein; they were developed to measure most α‐synuclein species present in CSF). The assays showed excellent within‐laboratory performance and round robin results showed that three out of four assays (Roche, ADx, and MSD) gave almost identical results in independent laboratories, whereas the BioLegend ELISA showed some inter‐laboratory variability, mostly driven by the results from one laboratory and likely attributable to variability with ELISA plate reader calibration, which has been observed with this assay during a multi‐laboratory comparison study in the past (Kruse *et al*. 2015).

When comparing the different assays to each other, it was clear that, although the correlations were excellent, they gave different absolute concentrations. The ADx and BioLegend assays in particular returned very similar values. Both assays were designed with two mAbs that recognize same epitopes on the protein. The most critical factor in standardization of results between assay designs might be related to how the assay calibration is done by each vendor/laboratory. Generally speaking, this type of outcome suggests that the assays measure similar forms of the biomarker and that they could be standardized to each other by the use of a common calibrator. This is where a certified reference material could be highly beneficial; commercial manufacturers may produce their own calibrators referenced to a common reference material. A prerequisite is that the reference material is commutable, meaning that it should have the same numeric relationship between different measurement procedures as representative CSF samples that one may encounter in clinical laboratory practice (Mattsson and Zetterberg 2012). Using the assay‐averaged samples as the common reference, the regression‐based harmonization methods (OLS and Passing‐Bablok) result in lower mean percent difference relative to the reference for assays MSD, BioLegend, and ADx compared to the single‐sample references (AL41, AL48). Noting the differing harmonization results obtained from the two single‐sample references, the choice of reference sample can have a non‐negligible impact on harmonization quality. For Roche, while the single‐sample references (AL48, AL41) actually lead to less deviation from the reference overall, the results are still worse than the regression‐based approaches used on the other three assays. Between the two regression approaches, Passing‐Bablok tends to have less deviation from the reference, especially at the low end, than OLS. Analytically, the Passing‐Bablok‐derived transformation harmonized the assay concentrations to the extent that all but one assay pair, BioLegend vs. Roche, was found to be commutable. The other three transformations did not perform as well. It needs to be mentioned, that the regression statistics comparing different methods to the mean of all the methods yield higher than expected values because of non‐independence of the data.

We also present pilot data on an IP‐MS method to quantify CSF α‐synuclein. Correlations to the immunoassays were good but the harmonization results using IP‐MS concentrations as reference were not as promising as those obtained with the average assay values as reference. As stated earlier, the regression‐based harmonization performs worse than the single‐sample reference methods for the Roche assay. Given the differences in technology, it is not unreasonable to assume that a nonlinear transformation (as opposed to the affine ones used here) would result in closer agreement with the reference. A potential drawback with the MS method used here to quantify α‐synuclein in CSF is that antibody enrichment was needed. However, the epitope is present and accessible in all (or most) α‐synuclein forms, and earlier data suggest that most α‐synuclein in a CSF sample is recovered (Schmid *et al*. 2018 in preparation). In any case, the IP‐MS‐based method represents an important step toward an MS‐based method for total α‐synuclein in CSF.

We are aware of the large inter‐individual variability in CSF α‐synuclein concentrations, which is not clearly understood. Other studies with larger cohorts have analyzed some factors, that showed no sex differences (Mollenhauer *et al*. 2011) and lower levels in PD subjects with tremor‐dominant phenotype in one study (Kang *et al*. 2013); other possible factors have been summarized in a previous review (Mollenhauer *et al*. 2016). This was beyond the scope of our study, which assessed analytical variation, but our data should be considered in this broader context.

Taken together, the results of this project show that several assays measure CSF α‐synuclein in a similar manner with excellent reproducibility and that it will be possible to standardize them to each other by the development of a reference material in which the concentration of α‐synuclein has been established using a reference method. Such a method should ideally have traceability to an agreed‐upon reference solution of the target analyte through an unbroken chain of comparisons. Work is currently on‐going to develop a mass spectrometry‐based candidate reference method for α‐synuclein. This would accelerate the adoption and incorporation of α‐synuclein assays into observational and interventional clinical trials as well as in preclinical studies. The standardization framework would also be useful once assays for pathology‐enriched forms of α‐synuclein have been developed.

## Acknowledgments and conflict of interest disclosure

This work was funded by the Michael J. Fox Foundation for Parkinson's Research Alpha‐Synuclein Assay Standardization LEAPS project. The authors would like to thank Kalpana M. Merchant, Poul Henning Jensen, and John Hale for their support and and advisement thoughout the project. The MRM analysis of human CSF was performed on a TSQ Vantage MS instrument, which was kindly funded by the ‘Roland Bailly Foundation’, Geneva, Switzerland. We thank the team from Roche Diagnostics International (namely, Veronika Corradini, Sebastian Dziadek and Richard Batrla‐Utermann) for including the Elecsys assay in this study and their helpful input. The study sponsors provided support through an unrestricted grant and had no influence on the study design, collection and analysis of data, the writing of the paper or the decision to submit the paper. The sponsors have been informed about the final manuscript and the submission for publication. Brit Mollenhauer: BM has received independent research grants from TEVA‐Pharma, Desitin, Boehringer Ingelheim, GE Healthcare and honoraria for consultancy from Bayer Schering Pharma AG, Roche, AbbVie, TEVA‐Pharma, Biogen and for presentations from GlaxoSmithKline, Orion Pharma, TEVA‐Pharma and travel costs from TEVA‐Pharma. BM is member of the executive steering committee of the Parkinson Progression Marker Initiative and PI of the Systemic Synuclein Sampling Study of the Michael J. Fox Foundation for Parkinson's Research and has received grants from the Deutsche Forschungsgemeinschaft, BMBF, EU (Horizon2020), Parkinson Fonds Deutschland, Deutsche Parkinson Vereinigung, Michael J. Fox Foundation for Parkinson's Research, Stifterverband für die deutsche Wissenschaft, and has scientific collaborations with Roche, Bristol Myers Squibb, Ely Lilly, Covance/BioLegend and Biogen. F. DuBois Bowman: No conflict of interest. Daniel Drake: No conflict of interest. Jimmy Duong: No conflicts of interest. Kaj Blennow: Kaj Blennow has served as a consultant or at advisory boards for Alzheon, BioArctic, Biogen, Eli Lilly, Fujirebio Europe, IBL International, Merck, Novartis, Pfizer, and Roche Diagnostics, and is a co‐founder of Brain Biomarker Solutions in Gothenburg AB, a GU Ventures‐based platform company at the University of Gothenburg. Omar El‐Agnaf: No conflict of interest. Leslie M Shaw receives research funding from the NIH/NIA, U19 AG024904 and P30AG010124; Michael J. Fox Foundation for Parkinson's Research; Eli Lilly; Hoffman LaRoche; served as a consultant for Eli Lilly, Hoffman LaRoche; has QC oversight for Roche Elecsys CSF AD biomarker immunoassays as part of the ADNI study. Jennifer Masucci is employed by BioLegend, San Diego, CA, USA. Peggy Taylor is employed BioLegend. Robert M. Umek is employed by Meso Scale Discovery. Jill M. Dunty is employed by Meso Scale Discovery. Chris L. Smith is employed by Meso Scale Discovery. Erik Stoops is employee and shareholder of ADx NeuroSciences. Hugo Vanderstichele Founder of Biomarkable, co‐founder of ADx NeuroSciences. Adrian W. Schmid: No conflict of interest. Marc Moniatte: No conflict of interest. Jing Zhang: No conflict of interest. Niels Kruse: No conflict of interest. Hilal A. Lashuel: HAL has received independent research grants from UCB, AC Immune and a PI and Co‐PI on several grants from the Michael J. Fox Foundation on biomarker discovery and assay development and standardization in Parkinson's disease. HLA has also served as a consultant for UCB and is a member of the scientific advisory board of Chaperone Therapeutics. Charlotte E. Teunissen received grants from the European Commission, the Dutch Research Council (ZonMW), Association of Frontotemporal Dementia/Alzheimer's Drug Discovery Foundation, Alzheimer Netherlands. Dr. Teunissen has functioned in advisory boards of Fujirebio and Roche, received non‐financial support in the form of research consumables from ADxNeurosciences and Euroimmun, performed contract research or received grants from Probiodrug, Janssen prevention center, Boehringer, Brainsonline, AxonNeurosciences, EIP farma, Roche. Tanja Schubert: No conflict of interest. Kuldip D. Dave is employed by the Michael J. Fox Foundation for Parkinson's Research; Samantha J. Hutten is employed by the Michael J. Fox Foundation for Parkinson's Research; Henrik Zetterberg has served at advisory boards of Eli Lilly and Roche Diagnostics, has received travel support from Teva, and is a co‐founder of Brain Biomarker Solutions in Gothenburg AB, a GU Ventures‐based platform company at the University of Gothenburg.

## Supporting information


**Figure S1.** Replicate Variation for Roche. Replicate variability (%CV) along y axis, study ID along × axis.
**Figure S2.** Replicate Variation for MSD. Replicate variability (%CV) along y axis, study ID along × axis.
**Figure S3.** Replicate Variation for BioLegend. Replicate variability (%CV) along y axis, study ID along × axis.
**Figure S4.** Replicate Variation for ADx. Replicate variability (%CV) along y axis, study ID along × axis.
**Figure S5**. Visualization of the kit lot‐averaged concentrations from each assay's originating site.
**Figure S6.** Immunoassay data harmonized to the average immunoassay concentrations.
**Figure S7.** Pairwise comparison of originating sites post‐harmonization.
**Figure S8.** Pairwise comparison of percent difference of Assay y relative to Assay x after harmonization to average immunoassay concentrations.
**Figure S9.** Immunoassay data harmonized to the IP‐MS average C‐Terminal fragment concentrations.
**Figure S10.** Pairwise comparison of originating sites post‐harmonization.
**Figure S11.** Pairwise comparison of percent difference of Assay y relative to Assay after harmonization to IP‐MS averaged C‐terminal measurements.
**Table S1.** Pairwise assay comparison post‐harmonization to the average immunoassay reference.
