# Supplementary material for: Antibody‐based methods for the measurement of α‐synuclein concentration in human cerebrospinal fluid – method comparison and round robin study
Source: J Neurochem. 2018 Nov 13;149(1):126–38. doi: 10.1111/jnc.14569 (PMC6587944; doi:10.1111/jnc.14569)

SUPPLEMENTARY INFORMATION

Antibody-based methods for the measurement of α-synuclein concentration in human cerebrospinal fluid - method comparison and round robin study

Brit Mollenhauer[1,2], MD; F. DuBois Bowman[3], PhD; Daniel Drake[3], PhD; Jimmy Duong[3], MPH; Kaj Blennow[4,5], MD; PhD; Omar El-Agnaf[6]; PhD; Leslie M. Shaw[7]; MD; Jennifer Masucci[8], BS; Peggy Taylor[8], ScD; Robert M. Umek[9], PhD; Jill M. Dunty[9], PhD; Chris L. Smith, PhD[9]; Erik Stoops[10], PhD, Hugo Vanderstichele[10], PhD; Adrian W. Schmid[11], PhD; Marc Moniatte, PhD[11]; PhD; Jing Zhang[12], MD; Niels Kruse[13], PhD; Hilal A. Lashuel[14], PhD; Charlotte Teunissen[15], PhD; Tanja Schubert[16], PhD; Kuldip D. Dave[17], PhD; Samantha J. Hutten[17], PhD; Henrik Zetterberg [4,5,18,19], MD

[1] Paracelsus-Elena-Klinik, Kassel, Germany
[2] University Medical Center, Department of Neurology, Göttingen, Germany
[3] Columbia University, Mailman School of Public Health, Department of Biostatistics, NYC, USA
[4] Department of Psychiatry and Neurochemistry, the Sahlgrenska Academy at the University of Gothenburg, Mölndal, Sweden
[5] Clinical Neurochemistry Laboratory, Sahlgrenska University Hospital, Mölndal, Sweden
[6] Neurological Disorders Research Center, Qatar Biomedical Research Institute (QBRI), and College of Science and Engineering, HBKU, Education City, Qatar Foundation, Doha, Qatar
[7] Department of Pathology & Laboratory Medicine and Center for Neurodegenerative Disease Research, Institute on Aging, University of Pennsylvania, Philadelphia, PA, USA
[8] BioLegend, Dedham, MA, USA
[9] Meso Scale Discovery, Gaithersburg, MD, USA
[10] ADx NeuroSciences, Gent, Belgium
[11] Proteomics Core Facility, Ecole Polytechnique Fédérale de Lausanne (EPFL), Lausanne, Switzerland
[12] University of Washington, Seattle, WA, USA
[13] University Medical Center, Institute of Neuropathology, Göttingen, Germany
[14] Laboratory of Molecular and Chemical Biology of Neurodegeneration, Brain Mind Institute, Institute of Physics of Biological Systems, Ecole Polytechnique Fédérale de Lausanne (EPFL), CH-1015, Lausanne, Switzerland
[15] VU University Medical Center, Amsterdam, the Netherlands
[16] Bioclinica, Lyon, France
[17] Michael J. Fox Foundation for Parkinson's Research, NYC, USA
[18] Department of Molecular Neuroscience, UCL Institute of Neurology, Queens Square, London, UK
[19] UK Dementia Research Institute at UCL, London, UK

Supplementary Table1.

Pairwise assay comparison post-harmonization to the average immunoassay reference. Four different harmonization transformations were tested (AL48, AL41, OLS, and Passing-Bablok). For each transformation, Passing-Bablok regression was performed to assess commutability of the various assays, augmented by the CUSUM test of linearity and Wald-Wolfowitz runs test, and Kendall's tau. Of the four transforms, the one derived from Passing-Bablok regression resulted in the most harmonized assay pairs: Roche vs. BioLegend was the only pair to fail both the CUSUM and Wald-Wolfowitz tests. All others were deemed harmonized based on Passing-Bablok intercept and slope 95% confidence intervals. Highlighted p-values for the CUSUM linearity test indicate a rejection of the null hypothesis that the two assays are linearly related. Highlighted p-values for the Wald-Wolfowitz indicate a rejection of the null hypothesis that the runs of positive and negative residuals about the regression line are randomly distributed. Light gray highlights for Passing-Bablok indicate that both intercept and slope confidence intervals straddle zero and one, respectively. Dark gray highlights indicate Passing-Bablok results that satisfy the confidence interval conditions, but for which the CUSUM linearity test failed.

				CUSUM			Passing-Bablok: Intercept			Passing-Bablok: Slope			Wald-Wolfowitz		
Harmonization Method	Assay X	Assay Y	Kendall tau	longest run	statistic	p	b	b LCI	b UCI	a	a LCI	a UCI	total runs	statistic	p
**AL48**	**MSD**	Bio Legend	0.81	3	0.61	0.84	-1637.00	-285.69	-69.26	1.13	1.03	1.26	30	1.46	0.19
		ADx	0.86	5	1.02	0.25	-13.00	-94.51	45.49	0.85	0.76	0.92	20	-1.46	0.19
		Roche	0.69	5	1.02	0.25	53.18	-61.23	149.94	0.84	0.72	0.98	17	-2.33	**0.03**
	**Bio Legend**	MSD	0.81	3	0.61	0.85	144.48	66.21	229.64	0.88	0.80	0.97	30	1.46	0.19
		ADx	0.86	3	0.61	0.85	109.30	44.86	162.87	0.74	0.67	0.83	29	1.17	0.31
		Roche	0.57	5	1.02	0.25	150.34	16.88	316.89	0.74	0.59	0.90	27	0.58	0.66
	**ADx**	MSD	0.86	5	1.02	0.25	15.34	-60.12	103.36	1.18	1.09	1.31	20	-1.46	0.19
		Bio Legend	0.86	3	0.61	0.85	-146.84	-239.72	-56.97	1.34	1.21	1.50	29	1.17	0.31
		Roche	0.63	4	0.82	0.52	20.67	-77.71	151.02	1.03	0.89	1.14	22	-0.88	0.47
	**Roche**	MSD	0.69	5	1.02	0.25	-63.25	-202.09	71.21	1.19	1.02	1.38	17	-2.33	**0.03**
		Bio Legend	0.58	5	1.02	0.25	-203.96	-519.67	-21.52	1.36	1.12	1.71	27	0.58	0.66
		ADx	0.63	4	0.82	0.52	-20.01	-171.47	70.73	0.97	0.88	1.14	22	-0.88	0.47
**AL41**	**MSD**	Bio Legend	0.81	3	0.61	0.85	-151.48	-263.64	-63.32	1.12	1.02	1.24	30	1.46	0.19
		ADx	0.86	5	1.02	0.25	-13.70	-99.50	48.27	0.95	0.85	1.03	20	-1.46	0.19
		Roche	0.69	6	1.22	0.10	52.80	-69.29	151.04	0.91	0.78	1.05	21	-1.17	0.31
	**Bio Legend**	MSD	0.81	3	0.61	0.85	135.03	62.96	213.39	0.89	0.80	0.98	30	1.46	0.19
		ADx	0.86	4	0.81	0.52	114.25	47.32	168.35	0.84	0.75	0.94	27	0.58	0.66
		Roche	0.57	5	1.02	0.25	151.73	16.66	319.83	0.80	0.63	0.98	23	-0.58	0.66
	**ADx**	MSD	0.86	5	1.02	0.25	14.47	-56.31	97.23	1.06	0.97	1.18	20	-1.46	0.19
		Bio Legend	0.86	4	0.82	0.52	-135.96	-224.99	-49.14	1.19	1.07	1.33	27	0.58	0.66
		Roche	0.63	4	0.82	0.52	22.89	-79.59	161.05	0.99	0.84	1.09	22	-0.88	0.47
	**Roche**	MSD	0.69	6	1.22	0.10	-58.26	-190.81	66.08	1.10	0.95	1.29	21	-1.17	0.31
		Bio Legend	0.57	5	1.02	0.25	-189.57	-475.56	-16.01	1.25	1.02	1.57	23	-0.58	0.66
		ADx	0.63	4	0.82	0.25	-23.12	-190.37	70.94	1.01	0.92	1.19	22	-0.88	0.47
**OLS**	**MSD**	Bio Legend	0.81	4	0.82	0.52	16.96	-75.29	90.41	0.98	0.89	1.08	26	0.29	0.89
		ADx	0.86	6	1.22	0.10	21.15	-65.26	81.08	0.99	0.89	1.08	20	-1.46	0.19
		Roche	0.69	6	1.22	0.10	-117.97	-268.27	-1.26	1.15	1.00	1.33	23	-0.58	0.66
	**Bio Legend**	MSD	0.81	4	0.82	0.52	-17.32	-100.16	69.97	1.02	0.92	1.12	26	0.29	0.89
		ADx	0.86	4	0.82	0.52	5.53	-74.86	69.06	1.01	0.90	1.12	27	0.58	0.66
		Roche	0.57	7	1.42	**0.03**	-183.45	-375.66	42.18	1.18	0.95	1.45	17	-2.33	**0.03**
	**ADx**	MSD	0.86	6	1.22	0.10	-21.32	-90.48	63.04	1.01	0.93	1.12	20	-1.46	0.19
		Bio Legend	0.86	4	0.82	0.52	-5.47	-76.89	67.28	0.99	0.89	1.11	27	0.58	0.66
		Roche	0.63	4	0.82	0.52	-190.78	-322.45	-27.67	1.19	1.03	1.32	20	-1.46	0.19
	**Roche**	MSD	0.69	6	1.22	0.10	102.19	0.76	207.66	0.87	0.75	1.01	23	-0.58	0.66
		Bio Legend	0.57	7	1.42	**0.03**	155.08	-46.37	266.83	0.85	0.69	1.05	17	-2.33	**0.03**
		ADx	0.63	4	0.82	0.52	159.67	25.79	248.90	0.84	0.75	0.98	20	-1.46	0.19
**PaBa**	**MSD**	Bio Legend	0.81	4	0.82	0.52	15.02	-80.24	88.60	0.98	0.89	1.09	26	0.29	0.89
		ADx	0.86	6	1.22	0.10	17.31	-69.16	77.05	0.99	0.89	1.08	20	-1.46	0.19
		Roche	0.69	6	1.22	0.10	-49.31	-186.48	60.32	1.09	0.93	1.26	23	-0.58	0.66
	**Bio Legend**	MSD	0.81	4	0.82	0.52	-15.34	-97.29	73.72	1.02	0.92	1.12	26	0.29	0.89
		ADx	0.86	4	0.82	0.52	3.69	-77.86	68.77	1.01	0.90	1.12	27	0.58	0.66
		Roche	0.57	7	1.43	**0.03**	-110.18	-294.76	106.81	1.12	0.89	1.38	17	-2.33	**0.03**
	**ADx**	MSD	0.86	6	1.22	0.10	-17.46	-85.71	67.30	1.01	0.93	1.13	20	-1.46	0.19
		Bio Legend	0.86	4	0.82	0.52	-3.66	-73.44	70.27	0.99	0.89	1.11	27	0.58	0.66
		Roche	0.63	4	0.82	0.52	-117.64	-241.63	38.53	1.14	0.98	1.27	18	-2.04	0.06
	**Roche**	MSD	0.69	6	1.22	0.10	45.12	-62.85	151.24	0.91	0.80	1.08	23	-0.58	0.66
		Bio Legend	0.57	7	1.43	**0.03**	98.26	-115.55	216.16	0.89	0.73	1.12	17	-2.33	**0.03**
		ADx	0.63	4	0.82	0.52	103.48	-35.68	196.07	0.88	0.79	1.02	18	-2.04	0.06

SUPPLEMENTARY FIGURES:

Supplementary Figure 1.
Replicate Variation for Roche. Replicate variability (%CV) along y axis, study ID along x axis.

Supplementary Figure 2.
Replicate Variation for MSD. Replicate variability (%CV) along y axis, study ID along x axis.

Supplementary Figure 3.
Replicate Variation for BioLegend. Replicate variability (%CV) along y axis, study ID along x axis.

Supplementary Figure 4.
Replicate Variation for ADx. Replicate variability (%CV) along y axis, study ID along x axis.

Supplementary Figure 5:
Visualization of the kit lot-averaged concentrations from each assay's originating site. The dotted line indicates the "mild" outlier threshold of 1.5 IQR above and below the third quartile. Under this definition, MSD has one mild outlier. A Grubbs' outlier test, though, fails to reject the null.

Supplementary Figure 6.
Immunoassay data harmonized to the average immunoassay concentrations. (a) plots immunoassay α-synuclein concentrations from the originating sites against the average assay concentrations. Four different methods were used to estimate the association between the individual assays and the reference: AL41 and AL48 denote fits that depend only on one sample; OLS (ordinary least squares) and PaBa (Passing-Bablok) take all 49 samples into account in their fitting procedure. All are characterized by their regression lines. (b) shows the results of transforming each assay based on its associated regression, bringing each

association with the reference close to the unit slope. Regression lines in this figure indicate results of Passing-Bablok regression on each of the four harmonized results. (c) indicates the percent difference between the harmonized assays and the reference.

Supplementary Figure 7.

Pairwise comparison of originating sites post-harmonization. Passing-Bablok regression provides estimates of slope and intercept. Line y = x (dotted) shown for reference.

Supplementary Figure 8.

Pairwise comparison of percent difference of Assay y relative to Assay x after harmonization to average immunoassay concentrations. Vertical axes for each assay have equal scale for ease of comparison.

Supplementary Figure 9.

Immunoassay data harmonized to the IP-MS average C-Terminal fragment concentrations. (a) plots immunoassay α-synuclein concentrations from the originating sites against the C-terminal of the IP-MS process. Three different methods were used to estimate the association between the individual assays and the reference: AL41 and AL48 denote fits that depend only on one sample; PaBa (Passing-Bablok) takes 48 samples into account in its fitting procedure (one sample, AL25 had negative α-synuclein concentration after harmonization and so was excluded). All three approaches are characterized by their regression lines. (b) shows the results of transforming each assay based on its associated regression, bringing each association with the reference close to the unit slope. Regression lines in this figure indicate results of Passing-Bablok regression on each of the four harmonized results. (c) indicates the percent difference between the harmonized assays and the reference.

Supplementary Figure 10.

Pairwise comparison of originating sites post-harmonization. Passing-Bablok regression provides estimates of slope and intercept. Line y = x (dotted) shown for reference.

Supplementary Figure 11.

Pairwise comparison of percent difference of Assay y relative to Assay after harmonization to IP-MS averaged C-terminal measurements. As before, vertical axes for each assay have equal scale for ease of comparison.

Supplementary Figure 1

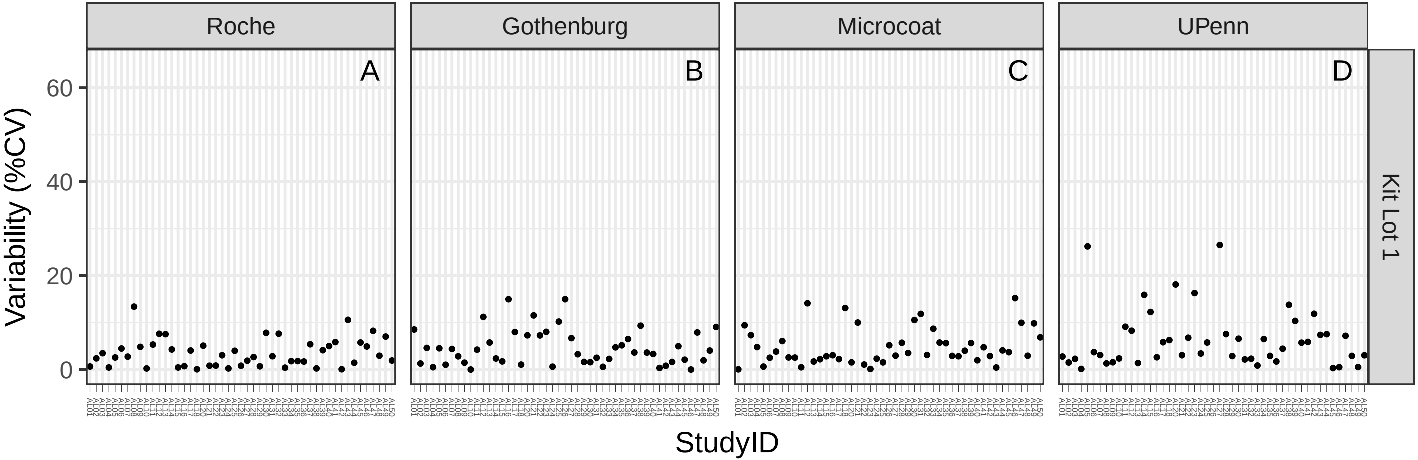

Supplementary Figure 2

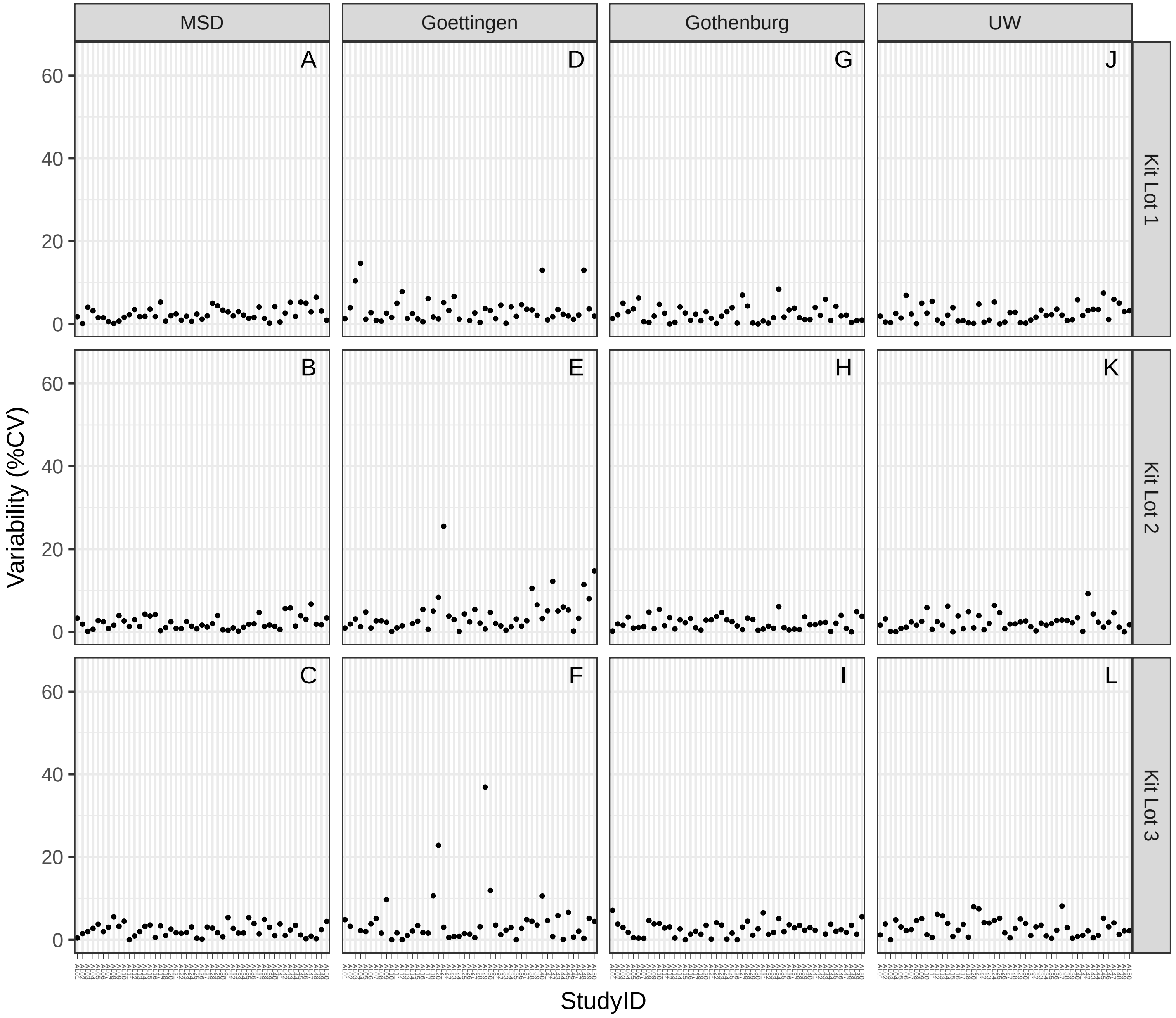

Supplementary Figure 3

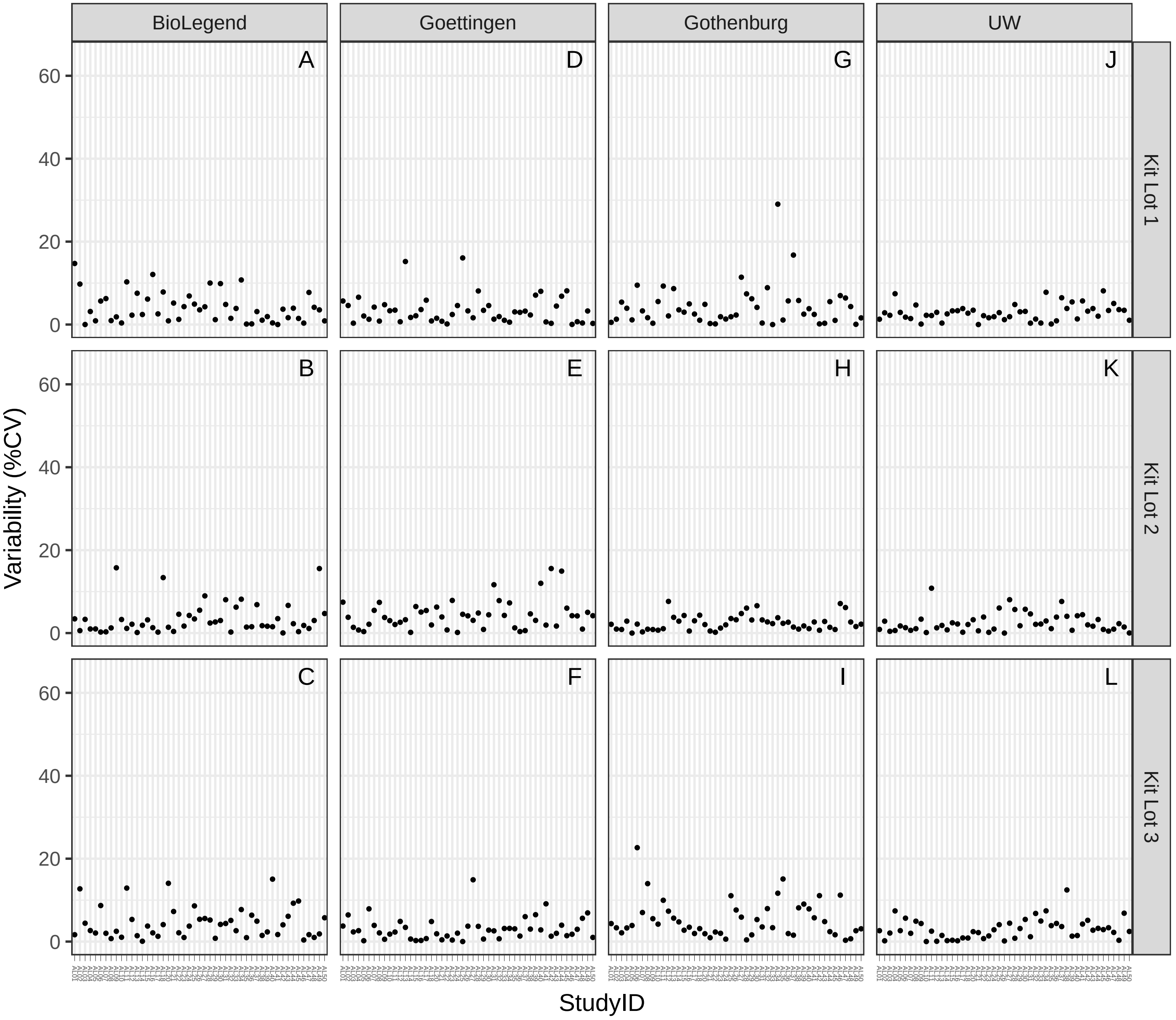

Supplementary Figure 4

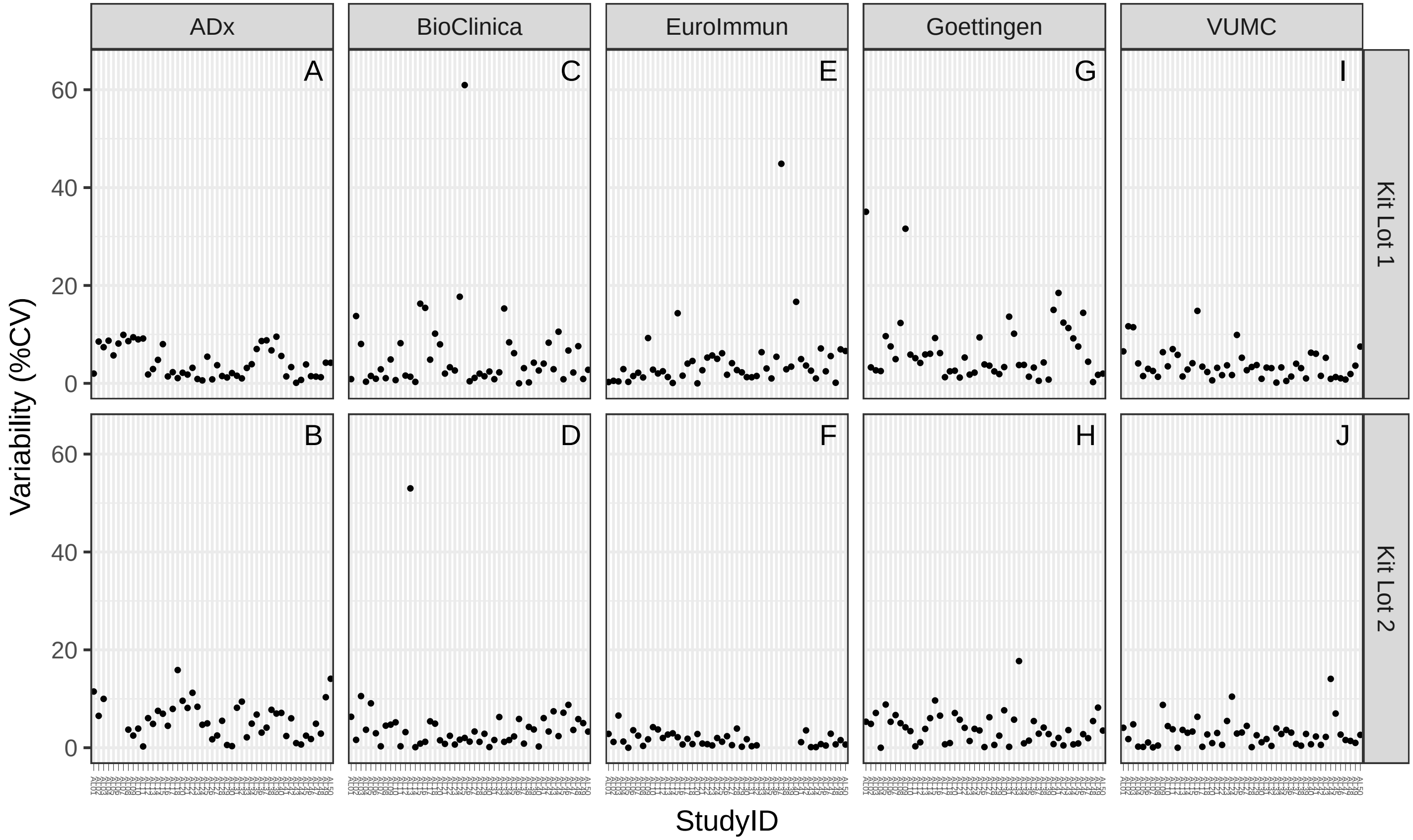

Supplementary Figure 5

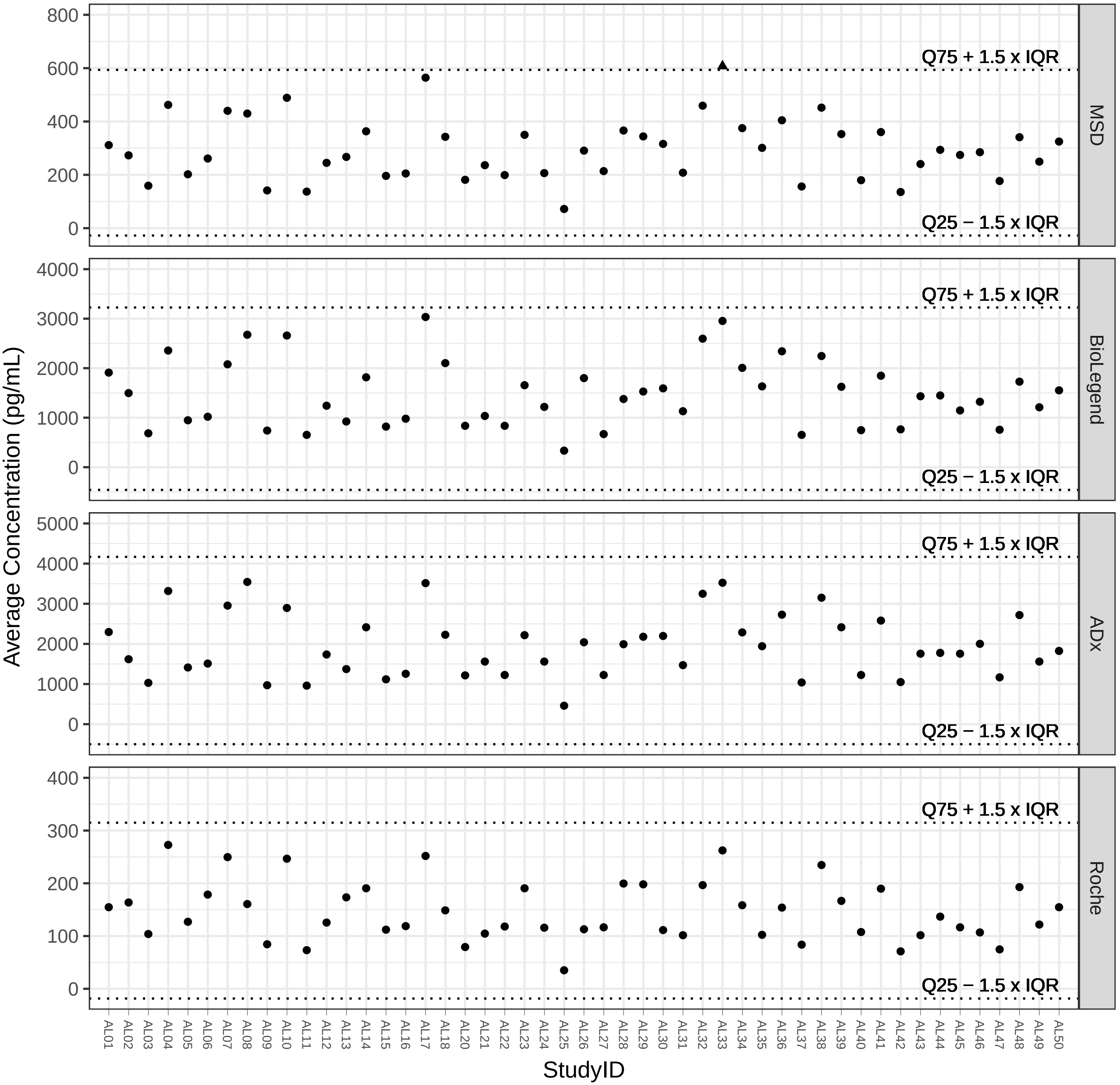

Supplementary Figure 6

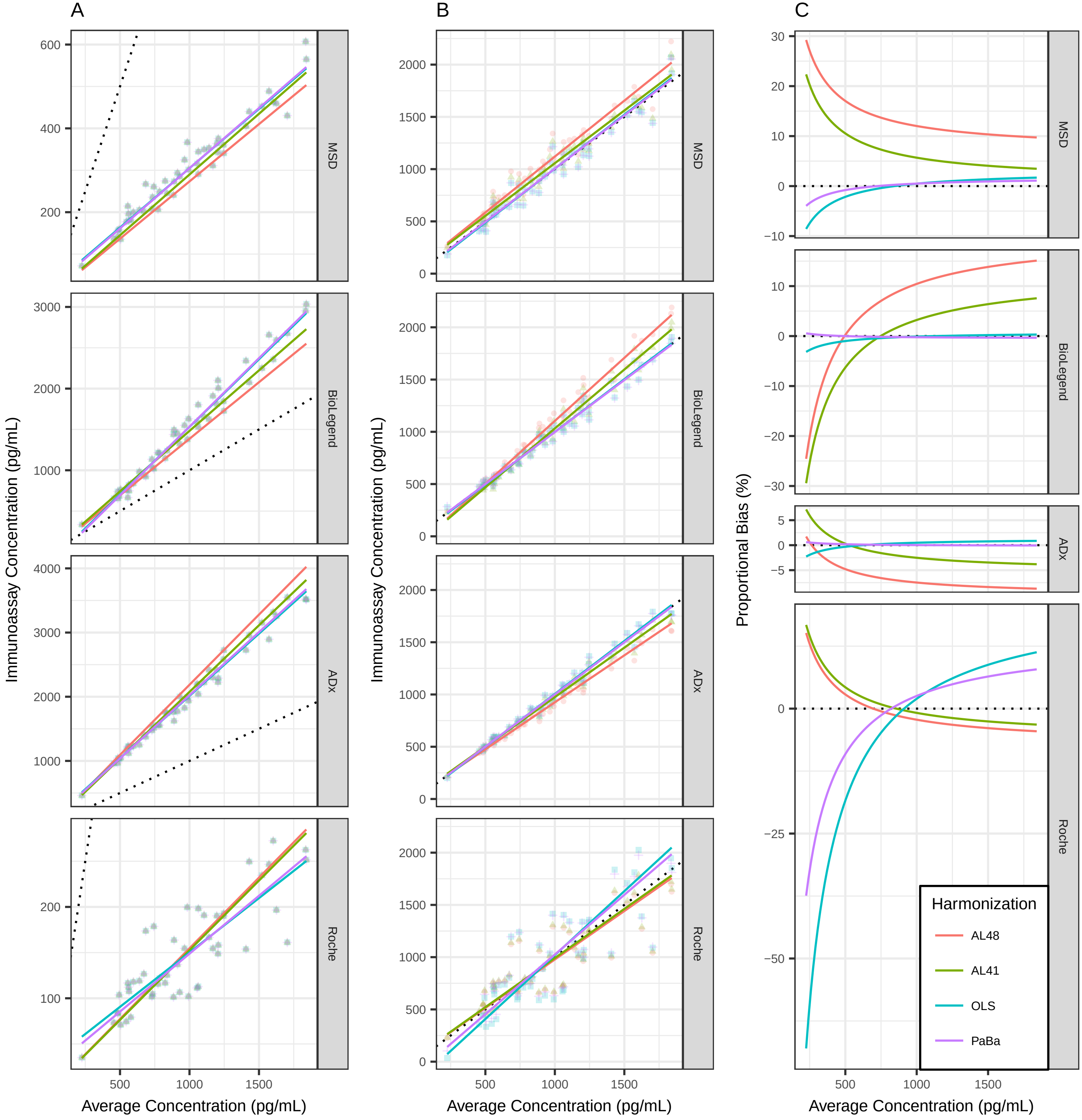

Supplementary Figure 7

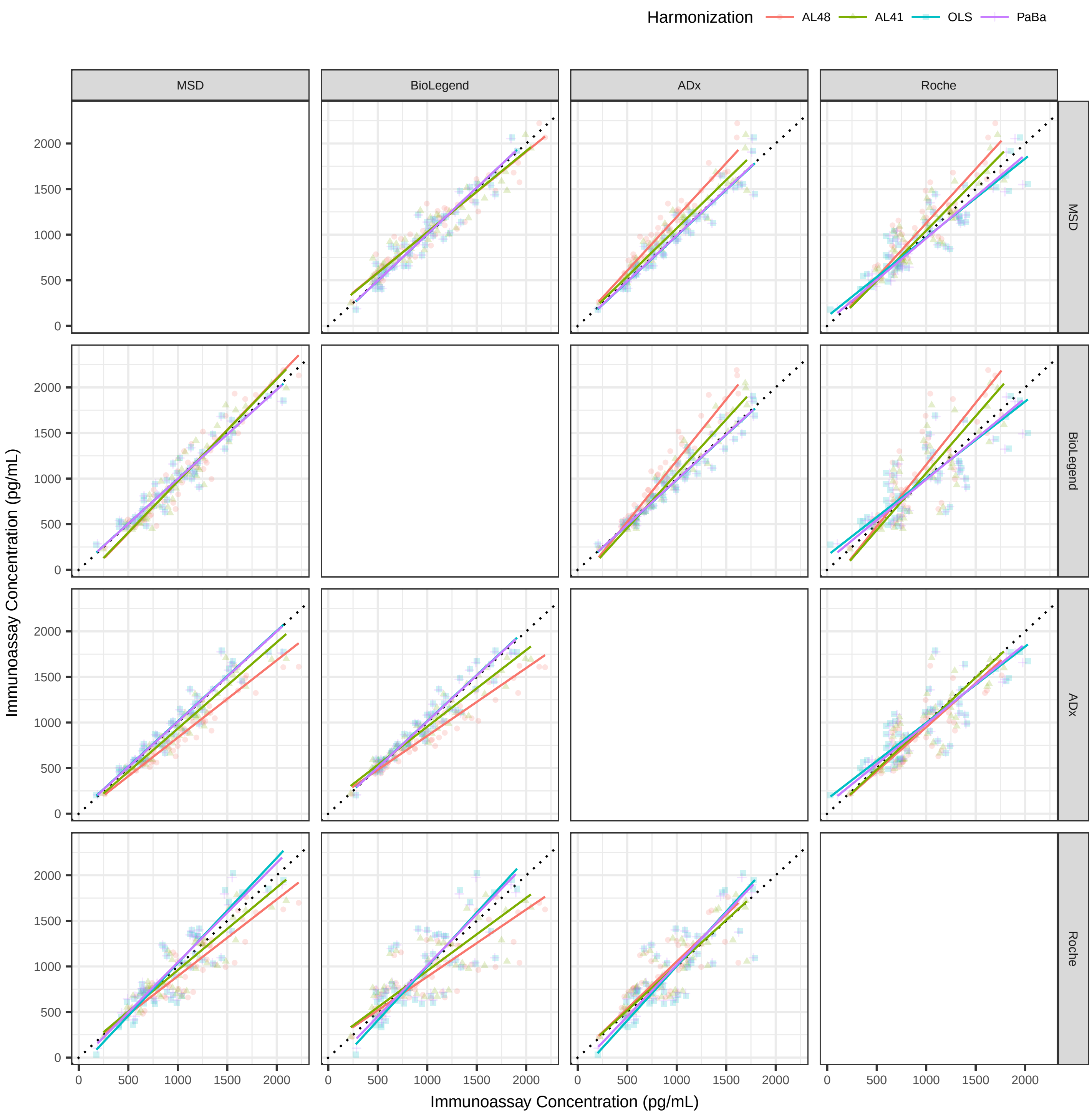

Supplementary Figure 8

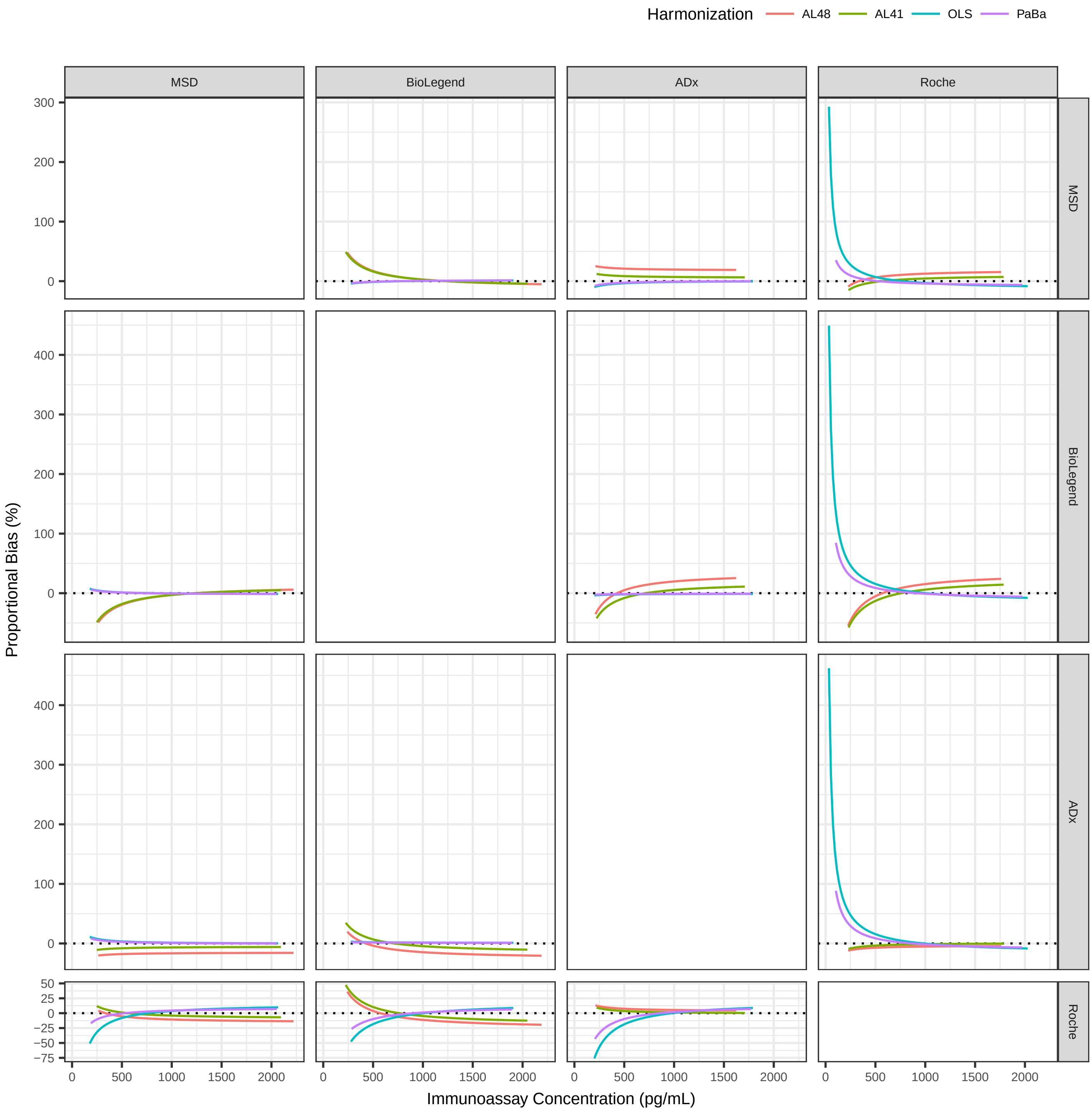

Supplementary Figure 9

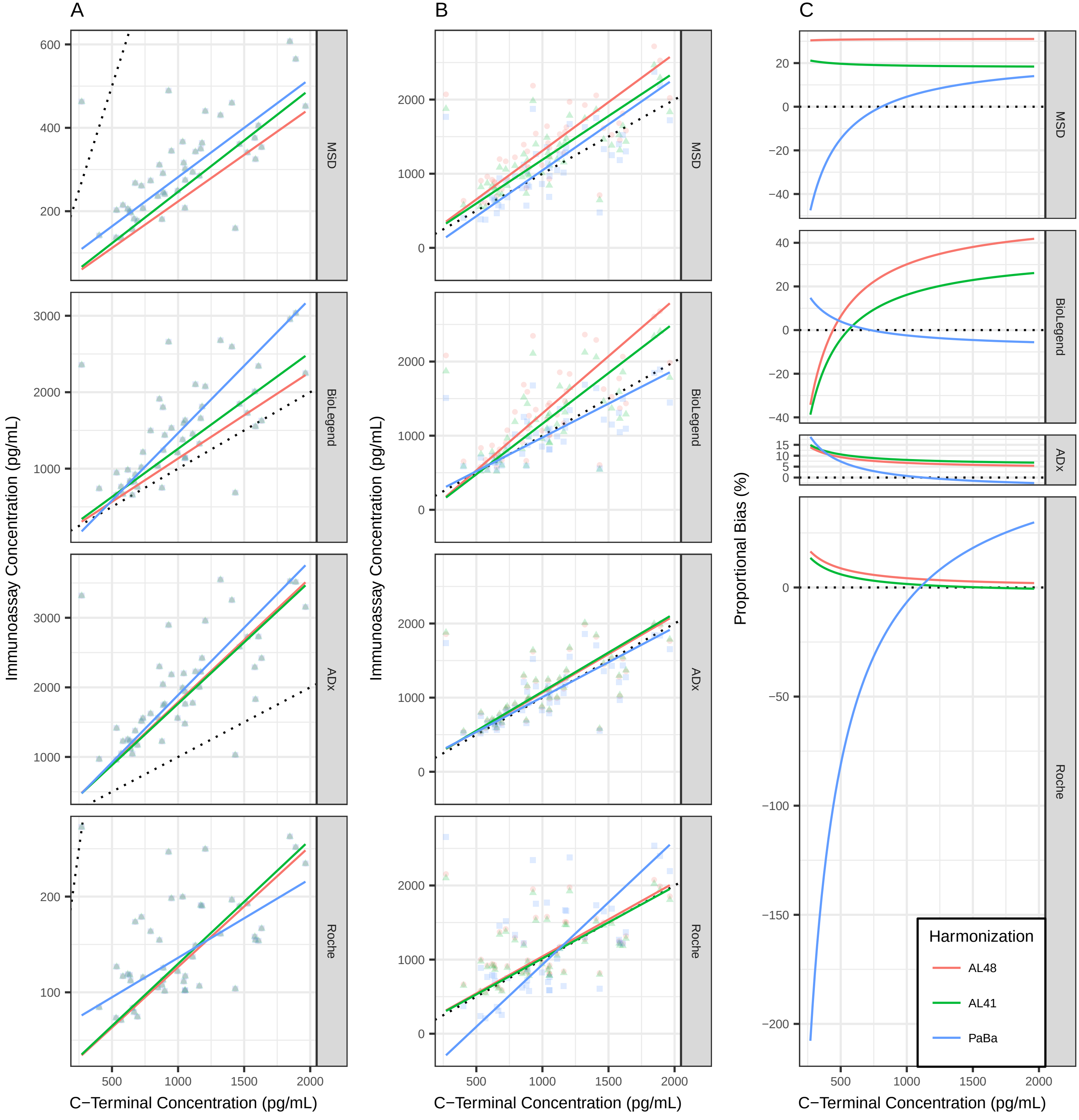

Supplementary Figure 10

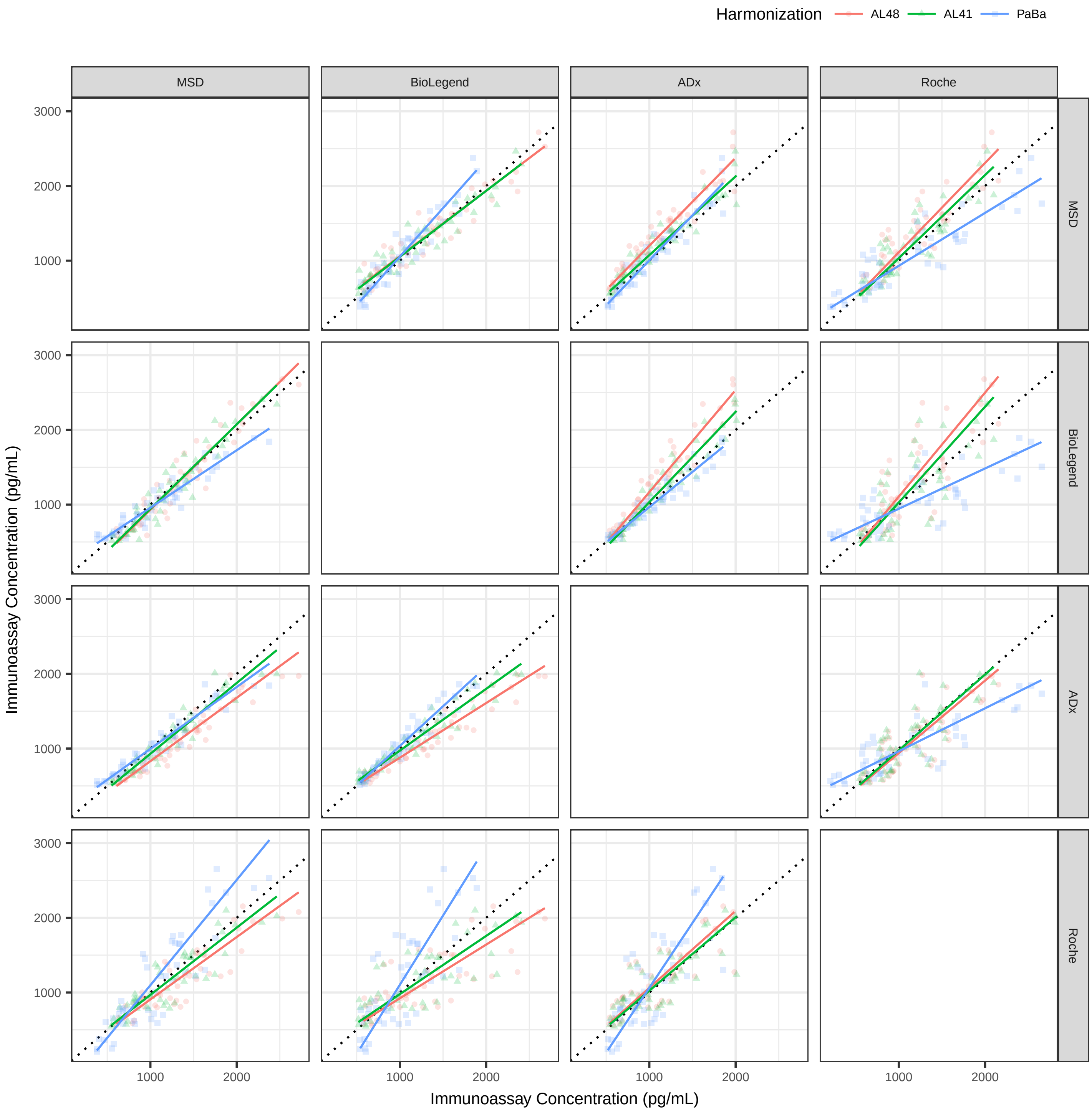

Supplementary Figure 11

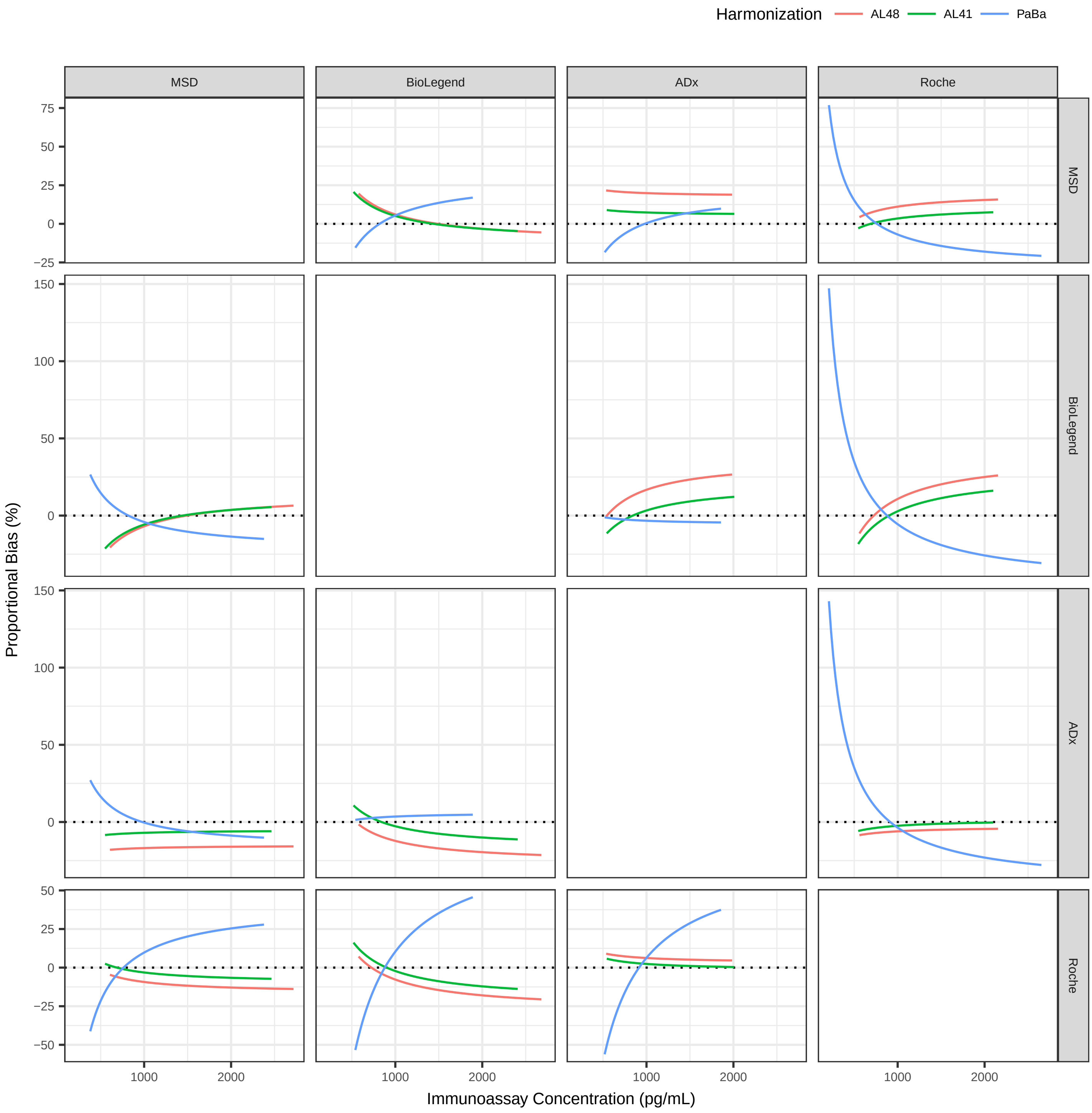

Supplement: Supplementary file 1 — Figure S1. Replicate Variation for Roche. Replicate variability (%CV) along y axis, study ID along × axis. Figure S2. Replicate Variation for MSD. Replicate variability (%CV) along y axis, study ID along × axis. Figure S3. Replicate Variation for BioLegend. Replicate variability (%CV) along y axis, study ID along × axis. Figure S4. Replicate Variation for ADx. Replicate variability (%CV) along y axis, study ID along × axis. Figure S5. Visualization of the kit lot‐averaged concentrations from each assay's originating site. Figure S6. Immunoassay data harmonized to the average immunoassay concentrations. Figure S7. Pairwise comparison of originating sites post‐harmonization. Figure S8. Pairwise comparison of percent difference of Assay y relative to Assay x after harmonization to average immunoassay concentrations. Figure S9. Immunoassay data harmonized to the IP‐MS average C‐Terminal fragment concentrations. Figure S10. Pairwise comparison of originating sites post‐harmonization. Figure S11. Pairwise comparison of percent difference of Assay y relative to Assay after harmonization to IP‐MS averaged C‐terminal measurements. Table S1. Pairwise assay comparison post‐harmonization to the average immunoassay reference. [file JNC-149-126-s001.pdf]
